# Liensinine inhibited gastric cancer cell growth through ROS generation and the PI3K/AKT pathway

**DOI:** 10.7150/jca.32691

**Published:** 2019-10-19

**Authors:** Jia-hua Yang, Kun Yu, Xian-ke Si, Sen Li, Yi-jun Cao, Wei Li, Ji-xun Zhang

**Affiliations:** Department of General Surgery, Putuo Hospital, Shanghai University of Traditional Chinese Medicine, 164 lanxi Rd, Shanghai, 200062, China.

**Keywords:** liensinine, gastric cancer, cell proliferation, ROS.

## Abstract

Liensinine, an isoquinoline alkaloid extracted from the seed embryo of Nelumbo nucifera Gaertn, has been shown to exhibit various phrenological effects, including anti‑cancer activity. The aim of this study is to investigate the effects and mechanisms of liensinine in human gastric cancer cells. In this study, we found liensinine can significantly inhibit gastric cancer cell proliferation *in vitro* and *in vivo*. Liensinine inducedgastric cancer cell apoptosis by increasing cleaved PARP, caspased 3 and caspased 9. Moreover, liensinine induced cycle arrest by downregulatingcyclinD1/cyclin‑dependent kinase4 and phosphorylated protein kinase B. Furthermore, we found liensinine increases ROS levels and inhibits the PI3K/AKT pathway. These data suggested that liensinine might represent a novel and effective agent against gastric cancer.

## Introduction

Gastric cancer(GC) is the fourth most common malignancy and the third leading cause of cancer mortality worldwide. In 2015, it was evaluated that GC was the second most common cancer in China, and the incidence of GC was two-fold higher in men than in women (320.8 vs 157.2 per 100,000), with the mean age being greater than 50^1^-^3^. There is still a pressing need to identify new prognostic biomarkers and therapeutic targets for this disease.

Nature-derived drugs, especially plant-derived compounds, have been widely used as therapeutic agents against cancer^4^. Traditionally, different parts of plants have been used for various medicinal purposes in Chinese traditional medicine. Liensinine, an isoquinoline alkaloid extracted from the seed embryo of Nelumbo nucifera Gaertn, has been shown to exhibit various phrenological effects^5^. Researchers found that liensinine can be used for treatment of arrhythmias, hypertension, pulmonary fibrosis and cancer. It was reported that liensinine could antagonize ventricular arrhythmias by blocking Ca2+ and Na+ influx^6^. Moreover, liensinine could block the binding of E2F1 at the transcription factor binding site of the FGFR2 promoter region and inhibit FGFR2 gene expression^7^. In addition, researchers found that liensinine could inhibit cancer tumorigenesis, including colorectal cancer and breast cancer. However, the effects of liensinine on gastric cancer have not been determined.

Reactive oxygen species (ROS) can activate intracellular signal transduction pathways in cancer, such as inflammation, cell cycle progression, apoptosis, migration and invasion^4^. A previous investigation showed that liensinine induced generation of ROS^8^. The induction of ROS by liensinine and the anti-cancer effects of liensinine on gastric cancer have not yet been illustrated.

In this study, we evaluated the antitumor effect of liensinine in human gastric cancer cells. We found that liensinineis able to inhibit proliferation of gallbladder cancer *in vitro* and *in vivo*. Furthermore, we demonstrated that liensinine could induce ROS generation and inhibit the PI3K/AKT pathway. This research provides a new approach for GBC therapeutic treatments.

## Materials and Methods

### Drugs and reagents

Liensinine was purchased from Shanghai Jinsui Bio-Technology Co., Ltd.; the purity was at least 99% as determined by high-performance liquid chromatography. Liensinine was dissolved in dimethyl sulfoxide (DMSO) and stored at -20 ℃. The final liensinine concentration did not exceed 0.1%. Cell Counting Kit-8 (CCK-8) was purchased from Beyotime Institute of Biotechnology Company (Suzhou, Jiangsu, China). Annexin V/Propidium Iodide (PI) Apoptosis Kit was purchased from Invitrogen (Carlsbad, CA, USA). All antibodies were purchased from Santa Cruz Biotechnology (Santa Cruz, CA, USA). All cell culture supplies were obtained from Invitrogen (Carlsbad, CA, USA).

### Cell culture

The BGC823, SGC7901, and GES-1 cells were purchased from the Cell Bank of the Type Culture Collection of the Chinese Academy of Sciences (Shanghai, Shanghai, China). All gastric cancer cells were cultured in RPMI 1640 with 10% (v/v) bovine calf serum. All cells were cultivated at 37℃ in a humidified incubator with 5% CO2.

### Cell viability assay

The CCK-8 assay was used to evaluate cell viability following the manufacturer's instructions. BGC823, SGC7901, and GES-1 cells were seeded into 96-well plates at a density of 5000 cells/well and were cultured for 24 h. Various concentrations of liensinine were added (0, 40, 60, 80, 100, and 120 μM) and incubated for 24 h, 48 h and 72 h. After each treatment, CCK-8 solution (10 μL) was added to each well and incubated for 2 h in the dark. A microplate reader (Bio-Tek, Norcross, GA, USA) was used to measure the absorbance at 450 nm. The IC50 values were determined using GraphPad Prism 5.

### Colony formation assay

BGC823 and SGC7901 cells were seeded into 6-well plates and treated with different concentrations of liensinine (0, 40, 60, and 80 μM). The cells were cultured for approximately 2 weeks. Then, the cells were fixed with 10% formalin and stained with 0.1% crystal violet (Sigma-Aldrich, St. Louis, MO, USA).

### Cell cycle analysis

PI staining and flow cytometry were used to analyze cell cycle distribution. We used different concentrations of liensinine (0, 40, 60, and 80 μM) to treat BGC823 and SGC7901 cells for 48 h. The cells were then washed by PBS (phosphate buffered saline) and fixed in 75% ethanol overnight at 4℃. Then, the cells were centrifuged and incubated with RNase (100 mg/mL) at 37℃ for 30 min. The cells were then stained with PI. Flow cytometry (FACS Calibur, BD, Bedford, MA, USA) was used to analyze the cellular DNA content and cell cycle distribution.

### Apoptosis analysis

An annexin V-FITC Apoptosis Detection Kit (BioVision, Milpitas, CA, USA) was used to carry out apoptosis analysis. BGC823 and SGC7901 cells were treated with different concentrations of liensinine for 48 h. Cells were collected by centrifugation and resuspended in 500 μL of 1 × binding buffer. Cells were then incubated with Annexin V-FITC (5 μL) and PI (5 μL) at 37 ℃ for 30 min. Cell apoptosis was analyzed by flow cytometry.

### Western blot analysis

BGC823 and SGC7901 cells were treated with different concentrations (0, 40, 60, and 80 μM) of liensinine for 48 h. Whole-cell cell lysates were separated using 10% SDS-PAGE gels and then transferred to PVDF membranes. The membranes were blocked and then probed with primary antibodies against corresponding antibodies overnight at 4℃. Next, the membranes were washed and incubated with appropriate secondary antibodies. Proteins were analyzed using a Gel Doc 2000 (Berkeley, California, USA).

### ROS detection

Intracellular ROS generation was assessed using an ROS assay kit according to the manufacturer's instructions.

### *In vivo* tumor xenograft study

Three to four week old BALB/c homozygous (nu/nu) nude mice with 18-20 g body weight were purchased from Shanghai SLAC Laboratory Animal Co Ltd. (Shanghai, Shanghai, China). The mice were housed in a specific pathogen-free environment with the temperature at 25℃±2℃ and a relative humidity of 70%±5%. SGC7901 cells (5×106) were suspended in 100 μL PBS and injected into the axilla of recipient mice. Twenty-four hours later, the mice were randomly divided into 2 groups (control and 10 μM). Mice received liensinine at the appropriate dose (0 and 10 μM) every 2 days for up to 1 month. Then, the animals were sacrificed, and their tumors were dissected and weighed.

## Results

### Subsection

#### Liensinine inhibits gastric cancer cell proliferation

**Figure 1:**
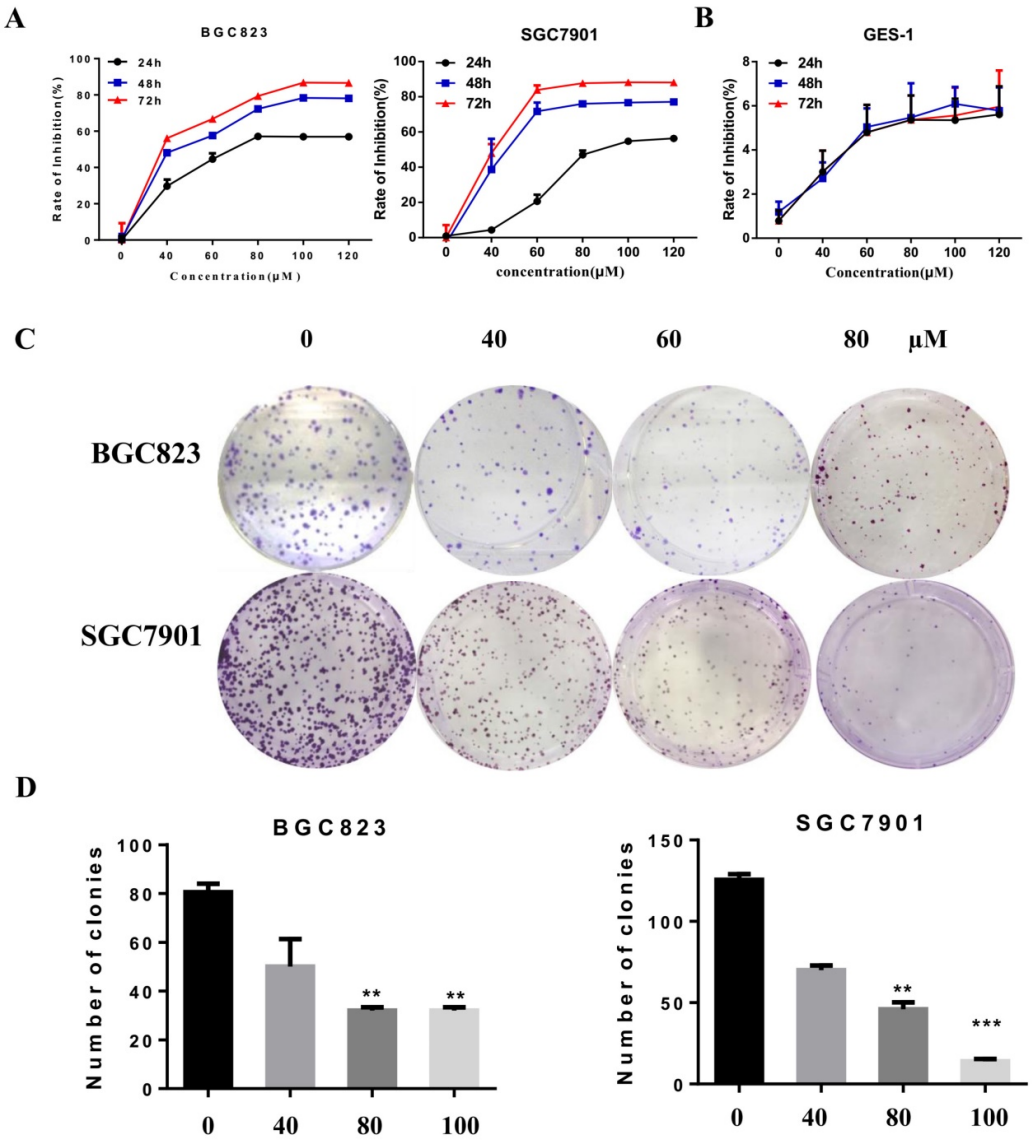
** Liensinine inhibits gastric cancer cell proliferation.** A-B) BGC823, SGC7901, and GES-1 cells were treated with various concentrations of liensinine for 24 h, 48 h, and 72 h. CCK8 assay was used to evaluate cell viability. C-D) Liensinine suppressed colony formation abilities of BGC823 and SGC7901 cells. Cells were treated with various concentrations of liensinine and cultured for 14 days to form colonies. The data are presented as the mean ± SD of three independent experiments. *P < 0.05, **P < 0.01, and ***P < 0.001 compared with the control.

To evaluate the inhibitory effect of liensinine on gastric cancer cell proliferation, CCK-8 assay and colony formation assays were performed. BGC823 and SGC7901 cells were exposed to increasing concentrations (0, 20, 40, 60, 80, 100, and 120μM) of liensinine for 24 h, 48 h, and 72 h, and the results showed that cell proliferation was significantly inhibited by DSN in a dose-dependent manner (Figure 1A).However, cell proliferation of normal gastric cell GES-1 was not inhibited (Figure 1B), which demonstrates the low toxic effect of liensinine.

The colony formation assay was used to detect the proliferation of a single cell. The BGC823 and SGC7901 cells were treated with various concentrations (0, 40, 60, and 80 µM) for approximately 2 weeks. As shown in Figure 1C-1D, the number and size of colonies derived from liensinine treated cells were markedly smaller compared with the control group. Collectively, these results suggest that liensinine exhibited a strong anti-proliferation effect in gastric cancer.

#### Liensinine induces gastric cancer cell apoptosis

**Figure 2:**
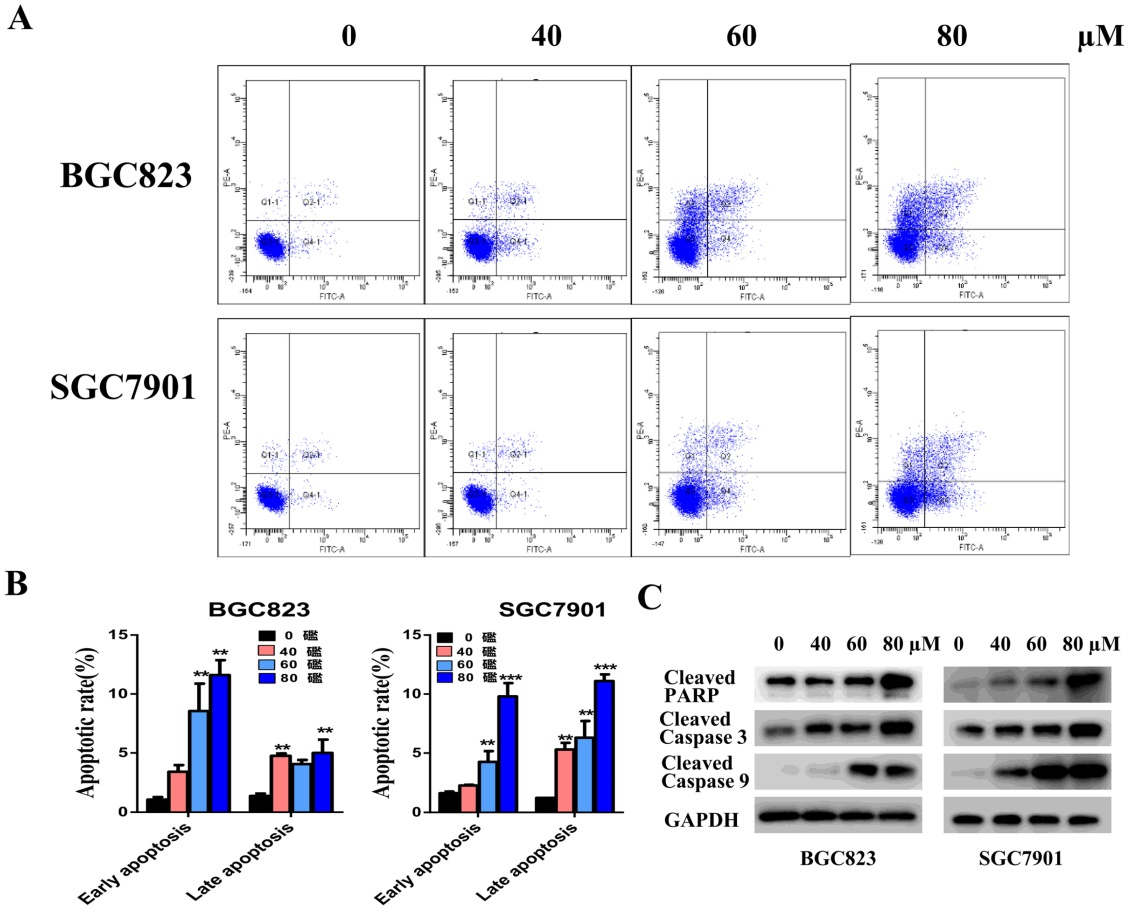
** Liensinine induces gastric cancer cell apoptosis.** A-B) BGC823 and SGC7901 cells were treated with various concentrations (0, 40, 60, and 80μM) of liensinine for 48 h, and apoptosis was evaluated by flow cytometry. C) The expression levels of cleaved caspase-3, cleaved caspase-9, and cleaved PARP were detected by western blot analysis.

We investigated the effect of casticin on apoptosis in gastric cancer cells using flow cytometry. BGC823 and SGC7901 cells were treated with different concentrations of liensinine (0, 40, 60, and 80 µM) for 48 h, and the percentages of apoptotic cells were determined by the Annexin V-FITC/PI staining assay by flow cytometry. Compared with the control group, the percentage of early and late apoptotic cells of liensinine-treated cells were strikingly elevated in a dose-dependent manner (Figure 2A-2B). Moreover, liensinine-treatment dramatically increased the expression of cleaved PARP, cleaved caspase 3, and cleaved caspase 9 (Figure 2C). The above data indicated that liensinine induces gastric cancer cell apoptosis.

#### Liensinine induces cell cycle arrest at G0/G1 phase

**Figure 3:**
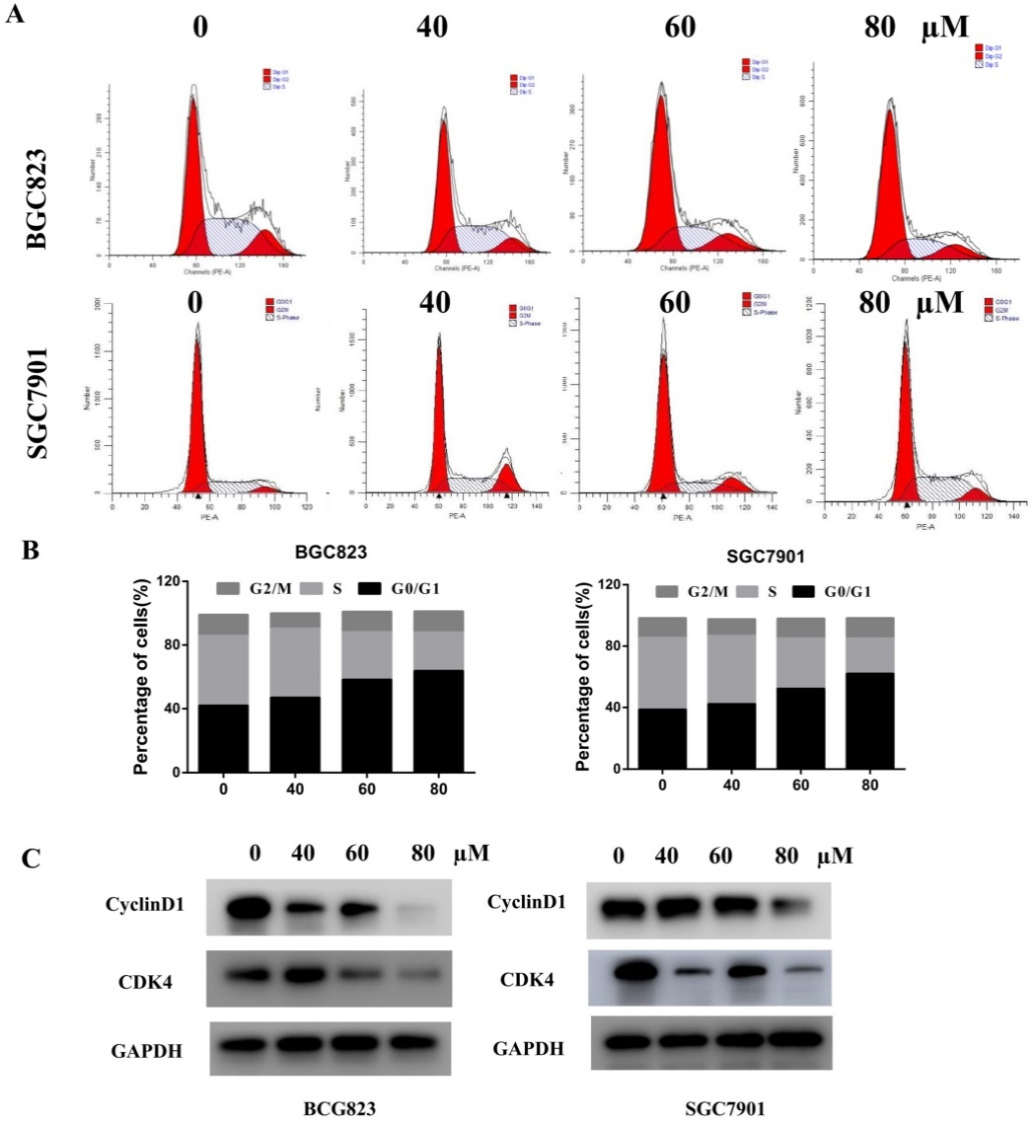
** Liensinine induces cell cycle arrest at the G0/G1 phase.** A-B) The cell cycle phases of treated cells were evaluated by flow cytometry. The x- axis represents the periodic distribution, and the y- axis represents the number of cells. C) The protein levels of cell cycle regulators, such CDK4 and cyclinD1, were examined by western blotting analysis. *P < 0.05, **P < 0.01, and ***P < 0.001 compared with the control.

To determine whether the growth-inhibitory effect of liensinine was mediated by cell cycle arrest, cell cycle distribution was examined by flow cytometry. The results indicated that the proportions of G0/G1 cells increased in a dose-dependent manner in BGC823 and SGC7901 cells, indicating that liensinine induced G0/G1 phase arrest (Figure 3A-3B). To examine the underlying mechanism of liensinine on cell cycle progression, we next analyzed several proteins that are involved in the regulation of G0/G1 progression by western blot analysis. Indicated by the results shown in Figure 3C, liensinine treatment resulted in decreased levels of cyclinD1 and CDK4, consistent with G0/G1 cell cycle arrest.

#### Liensinine increases ROS levels and inhibits the PI3K/AKT pathway in gastric cancer cells

**Figure 4:**
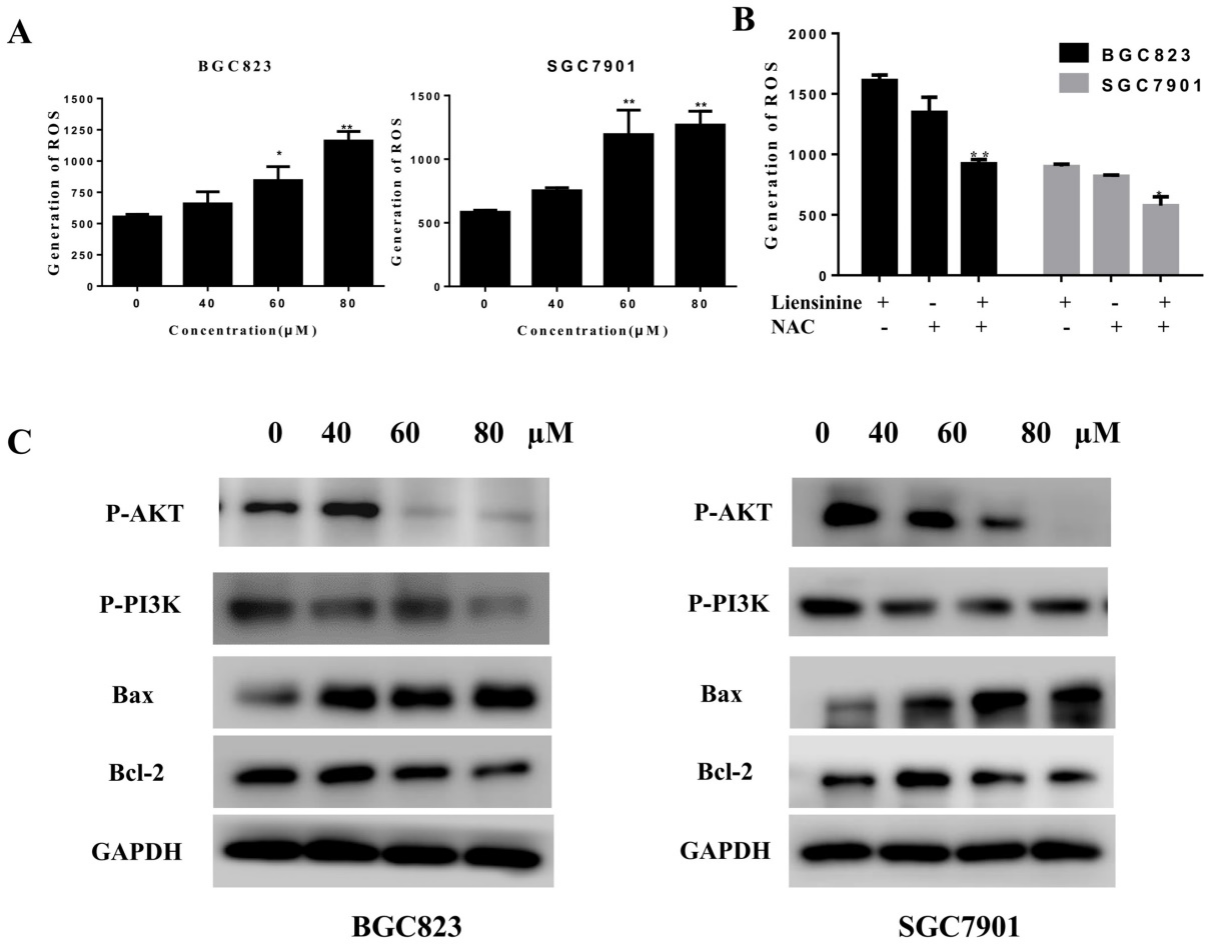
** Liensinine increases ROS levels and inhibits the PI3K/AKT pathway in gastric cancer cells.** A) BGC823 and SGC7901 cells were treated with various concentrations of liensinine for 48 h, followed by incubation with the fluorescent probe DCFH-DA (10μM) for 30 min, ROS generation was detected using a microplate reader. B) ROS generation were determined in the absence or presence of 10 mM NAC for 2h. C) The protein levels of P-PI3K and P-AKT were examined by western blotting analysis.

Many studies have revealed that ROS accumulation can induce cell death in many types of cancers after treatment with anti-cancer drugs^9^-^10^. Recent research found that the analogue of liensinine, isoliensinine could induce ROS accumulation in breast cancer cells^8^. Therefore, we investigated whether ROS are associated with liensinine-induced apoptosis. We used fluorescent probe (DCFH-DA) to monitor the intracellular ROS level with different concentrations of liensinine (0, 40, 60, and 80 µM) for 48 h. Indicated by the results in Figure 4A, increasing amounts of ROS were generated in a dose-dependent manner in BGC823 and SGC7901 cells. Next, we pretreated cells with or without 10 mM NAC for 2 h and then subjected cells to liensinine for an additional 48 h; as expected, NAC abolished liensinine-induced ROS accumulation (Figure 4B). Collectively, our results showed that ROS accumulation was involved in liensinine-induced apoptosis.

Many anticancer compounds induce ROS formation and inhibit the PI3K/AKT pathway, and ultimately cause apoptosis in cancer cells^4^,^11^-^15^. We estimated the effects of liensinine on PI3K/AKT-related protein expression by western blot analysis. As shown in Figure 4C, liensinine significantly inhibited the expression of p-AKT, p-PI3K, and Bcl-2, and increased the expression of Bax. These results indicate that the PI3K/AKT signaling pathway is associated with liensinine-induced apoptosis.

#### Liensinine inhibits tumor growth *in vivo*

**Figure 5:**
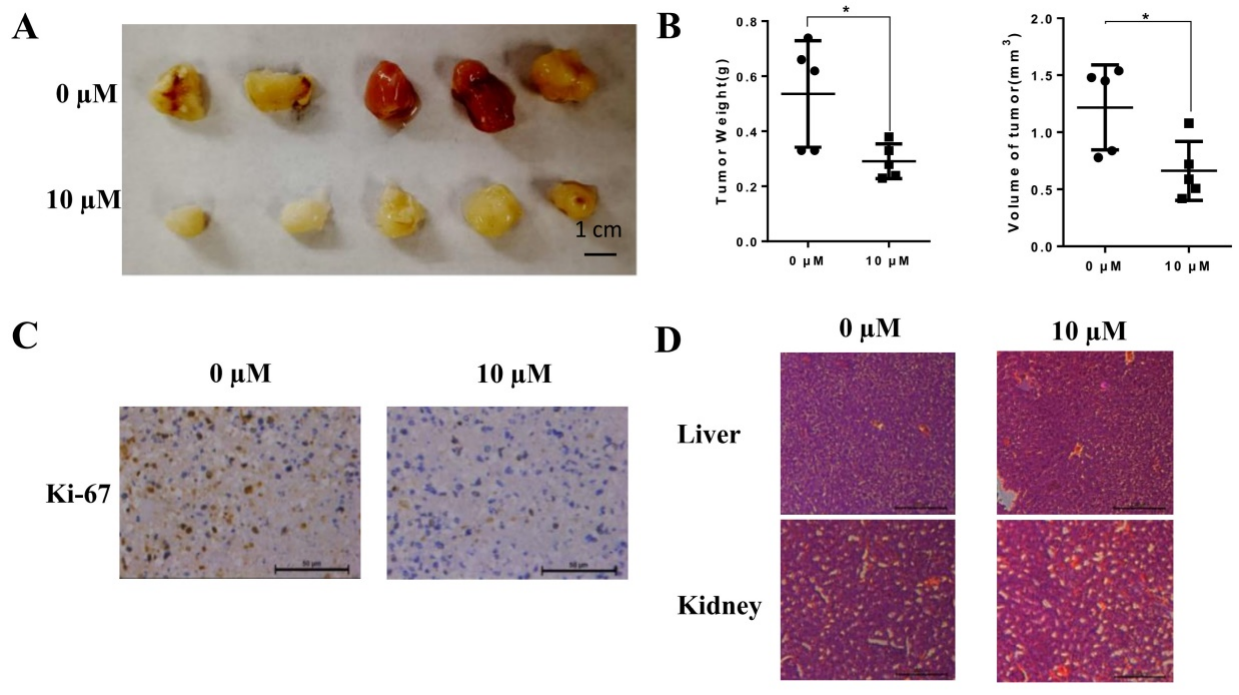
** Liensinine inhibits tumor growth *in vivo*.** A) Different concentrations (0 and 10 µM) of liensinine were injected into nude mice every 2 days. The weights and volumes of the tumors were measured. B) IHC staining of ki-67 of different concentrations of liensinine. C) HE staining of the mice's livers and spleens.

To evaluate the anti-cancer effects of liensinine *in vivo*, we injected mice with liensinine at a concentration of 0 and 10 µM every 2 days after inoculation^16^. The results showed that the tumor burden was markedly inhibited by liensinine (Fig. 5A-5B). Based on this observation, we performed HE staining and IHC analysis. As shown in Figure 5C, ki-67 expression levels were significantly reduced compared with those of the control group. Moreover, from HE staining of the mice's liver and spleen, liensinine showed no significant effect on organ structures compared to the control group (Figure 5D). These results are consistent with the *in vitro* effects of liensinine.

## Discussion

Traditional Chinese medicine has been extensively used to treat various diseases for thousands of years. As a bioactive compound of the Nelumbo nucifera, liensinine is generally recognized as safe for use in daily food, additionally; more and more evidence has shown the liensinine exhibits a potential anti-cancer effect, Wang et al found that liensinine perchlorate inhibits colorectal cancer tumorigenesis by inducing mitochondrial dysfunction and apoptosis. Zhou et al found that liensinine sensitizes breast cancer cells to chemotherapy through DNM1L-mediated mitochondrial fission. 17-18. In our study, we explored the anti-cancer effect of liensinine and its potential mechanism.

In this study, CCK-8 and colony formation assays were performed to assess proliferation and viability of gastric cancer cells with liensinine treatment. We found that liensinine significantly reduced the proliferation of gastric cancer cells in a dose-dependent manner, but was not highly toxic to normal gastric cells even at higher doses (100 μM). In the following, we evaluated the effects of liensinine treatment in mice with xenografted tumors. Based on the weights and volumes, HE staining of livers and spleen of two groups, we concluded that liensinine exerted anti-cancer effects on gastric cancer cells *in vivo*. Our results also confirmed that liensinine induced apoptosis in gastric cancer cells.

The mammalian cell cycle is controlled by a series of highly regulated processes, and its dysregulation is a hallmark of human cancer^19^-^21^. Progression through the cell cycle depends on the activation of cyclin-dependent kinases (CDKs) and their regulatory subunits, the cyclins^22^. In the present study, we found that treatment with liensinine could induce gastric cancer cells to arrest at the G0/G1 phase. To further confirm the effect of liensinine on cell cycle phase, we performed a western blot analysis. We confirmed the result that liensinine could induce G0/G1 arrest.

Normally, ROS can function as signals to regulate cell proliferation and survival, whereas a disproportionate amount of ROS can damage cellular components, disturb normal cellular processes and lead to cell death^23^ .Evidence has suggested that liensinine and its analogue exhibit potential anti-cancer effects. Neferine, an analogue of isoliensinine, inhibited high glucose-induced apoptosis in endothelial cells by blocking ROS generation^8^. Liensinine could also induce mitochondrial-mediated ROS generation in triggering apoptosis in HepG2 cells. In our study, we found that ROS increased with increasing concentration treatments of liensinine. Moreover, to investigate whether ROS play a potential role, ROS scavenger NAC could significantly attenuate apoptosis induced by liensinine in gastric cancer cells. Above all, we believe that ROS was involved in apoptosis of gastric cancer.

PI3K/AKT has been proved to be a common downregulated signaling pathway in human cancers. Previous studies reported that the activation of the PI3K/AKT pathway can induce cell growth^22^,^24^-^25^. In our study, as the results of western blot analysis have shown, the expressions of P-AKT and P-PI3K in gastric cancer cells were significantly reduced by liensinine. We thus confirmed that liensinine induced apoptosis by inhibiting the PI3K/AKT signaling pathway. The PI3K/Akt pathway acts as a pivotal determinant of cell biology and disease progression including cancer and aging, which is the vital downstream of ROS 26.Song et al found that dioscin could induced gallbladder cancer cells apoptosis via ROS-induced PI3K/AKT pathway^4^. In our study, we also found liensinine could induce gastric cancer cells apoptosis by ROS accumulation and PI3K/AKT signaling pathway. However, the direct link between ROS and the PI3K/AKT pathway should be further investigated in future studies.

In summary, the present study showed that liensinine exhibits an anti-cancer effect in gastric cancer cells. Its anti-cancer effect was achieved by inducing ROS accumulation and by inhibiting the PI3K/AKT signaling pathway. Therefore, we believe that liensinine may be a novel and effective therapy for gastric cancer.
